# FAK Regulates Intestinal Epithelial Cell Survival and Proliferation during Mucosal Wound Healing

**DOI:** 10.1371/journal.pone.0023123

**Published:** 2011-08-24

**Authors:** Katherine A. Owen, Michelle Y. Abshire, Robert W. Tilghman, James E. Casanova, Amy H. Bouton

**Affiliations:** 1 Department of Cell Biology, University of Virginia Health System, Charlottesville, Virginia, United States of America; 2 Department of Microbiology, University of Virginia Health System, Charlottesville, Virginia, United States of America; Emory University, United States of Amerca

## Abstract

**Background:**

Following damage to the intestinal epithelium, restoration of epithelial barrier integrity is triggered by a robust proliferative response. In other tissues, focal adhesion kinase (FAK) regulates many of the cellular processes that are critical for epithelial homeostasis and restitution, including cell migration, proliferation and survival. However, few studies to date have determined how FAK contributes to mucosal wound healing *in vivo*.

**Methodology and Principal Findings:**

To examine the role of FAK in intestinal epithelial homeostasis and during injury, we generated intestinal epithelium (IE)-specific conditional FAK knockout mice. Colitis was induced with dextran-sulfate-sodium (DSS) and intestinal tissues were analyzed by immunohistochemistry and immunoblotting. While intestinal development occurred normally in mice lacking FAK, FAK-deficient animals were profoundly susceptible to colitis. The loss of epithelial FAK resulted in elevated p53 expression and an increased sensitivity to apoptosis, coincident with a failure to upregulate epithelial cell proliferation. FAK has been reported to function as a mechanosensor, inducing cyclin D1 expression and promoting cell cycle progression under conditions in which tissue/matrix stiffness is increased. Collagen deposition, a hallmark of inflammatory injury resulting in increased tissue rigidity, was observed in control and FAK knockout mice during colitis. Despite this fibrotic response, the colonic epithelium in FAK-deficient mice exhibited significantly reduced cyclin D1 expression, suggesting that proliferation is uncoupled from fibrosis in the absence of FAK. In support of this hypothesis, proliferation of Caco-2 cells increased proportionally with matrix stiffness *in vitro* only under conditions of normal FAK expression; FAK depleted cells exhibited reduced proliferation concomitant with attenuated cyclin D1 expression.

**Conclusions:**

In the colon, FAK functions as a regulator of epithelial cell survival and proliferation under conditions of mucosal injury and a mechanosensor of tissue compliance, inducing repair-driven proliferation in the colonic epithelium through upregulation of cyclin D1.

## Introduction

The intestinal epithelium serves as a selective permeability barrier, separating the intestinal lumen and its contents from underlying tissues [1]. Breach of this mucosal barrier puts the host at risk for infection and inflammation, thus requiring a rapid and efficient response to injury. The restoration of tissue integrity involves the coordinated interaction of various cell types, deposition of extracellular matrix (ECM), release of soluble growth factors, and upregulation of epithelial cell proliferation [1], [2].

Adhesion-mediated signaling between cells and the ECM plays a critical role in maintaining tissue homeostasis as well as in the response to tissue damage [1]. Focal adhesion kinase (FAK) is a non-receptor tyrosine kinase that is involved in adhesion signaling in multiple cell types, including those of epithelial derivation. Through its kinase activity, FAK provides robust, anti-apoptotic signals involving the PI3K/Akt and MAPK pathways [3]. Expression of dominant-negative FAK mutants in intestinal epithelial cell lines leads to increased apoptosis due to the loss of adhesion-mediated survival signals [4], [5]. Conversely, FAK over-expression has been shown to suppress apoptosis by activating the nuclear factor kappa B (NF-kB) pathway [6]. FAK also promotes cell survival by binding to, and inducing the degradation of, the tumor suppressor protein p53. The induction of cellular stress through DNA damage, hypoxia and/or onocogene activation induces p53-mediated transcription of genes involved in cell death and cell cycle arrest, while at the same time inhibiting the transcription of cell survival genes [7], [8]. Under these conditions, FAK promotes cell survival by entering the cell nucleus and causing the degradation of p53 [9].

In addition to its role mediating cell survival, FAK has also been shown to regulate cellular proliferation. In one mechanism, FAK autophosphorylation at tyrosine 397 creates a binding site for Src family kinases, which in turn promotes Src-dependent tyrosine phosphorylation of FAK at other sites [10]. The adaptor molecule Grb2 binds to phosphorylated tyrosine 925, initiating the Ras/MEK/ERK signaling cascade and activation of Ets-like transcription factors that promote cyclin D1 expression and progression through the cell cycle [10], [11]. Independent of ERK activation, FAK regulates a second transcription factor, Krupple-like factor 8 (KLF8), which binds to and upregulates the cyclin D1 promoter [12]. Finally, FAK can function as a mechanosensor of tissue rigidity, promoting proliferation in response to decreased tissue compliance via the upregulation of cyclin D1 [13].

In this study, we investigated the role of FAK in intestinal development and colonic injury using an intestinal epithelial (IE)-conditional FAK knockout mouse model in which FAK is deleted from both the small and large intestine. Loss of FAK in these mice had no significant effect on intestinal development or function under homeostatic conditions. However, colonic epithelial repair was significantly impaired in the absence of FAK following inflammatory injury induced by acute dextran sulfate sodium (DSS) treatment. Mice lacking FAK exhibited earlier onset and increased severity of disease relative to control animals, characterized by more extensive edema, ulceration and disruption of crypt architecture. Upon removal of DSS, control mice exhibited rapid epithelial restitution and a coincident increase in epithelial cell proliferation. Conversely, DSS treatment resulted in the accumulation of p53 in FAK-deficient epithelial cells and increased evidence of apoptosis as measured by activation of caspase-3. In addition, proliferation was significantly impaired in the FAK-deficient mice and this correlated with a reduction in cyclin D1 levels, coincident with a failure to repair the epithelium.

Collagen deposition is a hallmark of inflammatory injury, and has been reported to induce tissue stiffening (fibrosis) in inflammatory bowel disease [14], [15]. As discussed above, FAK functions as a mechanosensor of matrix rigidity and has been shown to promote cell proliferation in response to increased tissue stiffness by inducing cyclin D1 expression [13]. While collagen deposition was observed in the colon following DSS treatment in both WT and FAK-deficient animals, epithelial cyclin D1 expression was elevated only in control mice. A similar loss of sensitivity to matrix stiffness and reduced cyclin D1 levels were observed in Caco-2 intestinal epithelial cells depleted of FAK by RNA interference. These findings suggest that FAK functions *in vivo* both as a regulator of adhesion-mediated survival and proliferation, as well as a mechanotransducer of tissue compliance required to drive cell cycle progression in response to inflammatory injury.

## Results

### FAK is dispensable for normal intestinal development

**Figure 1:**
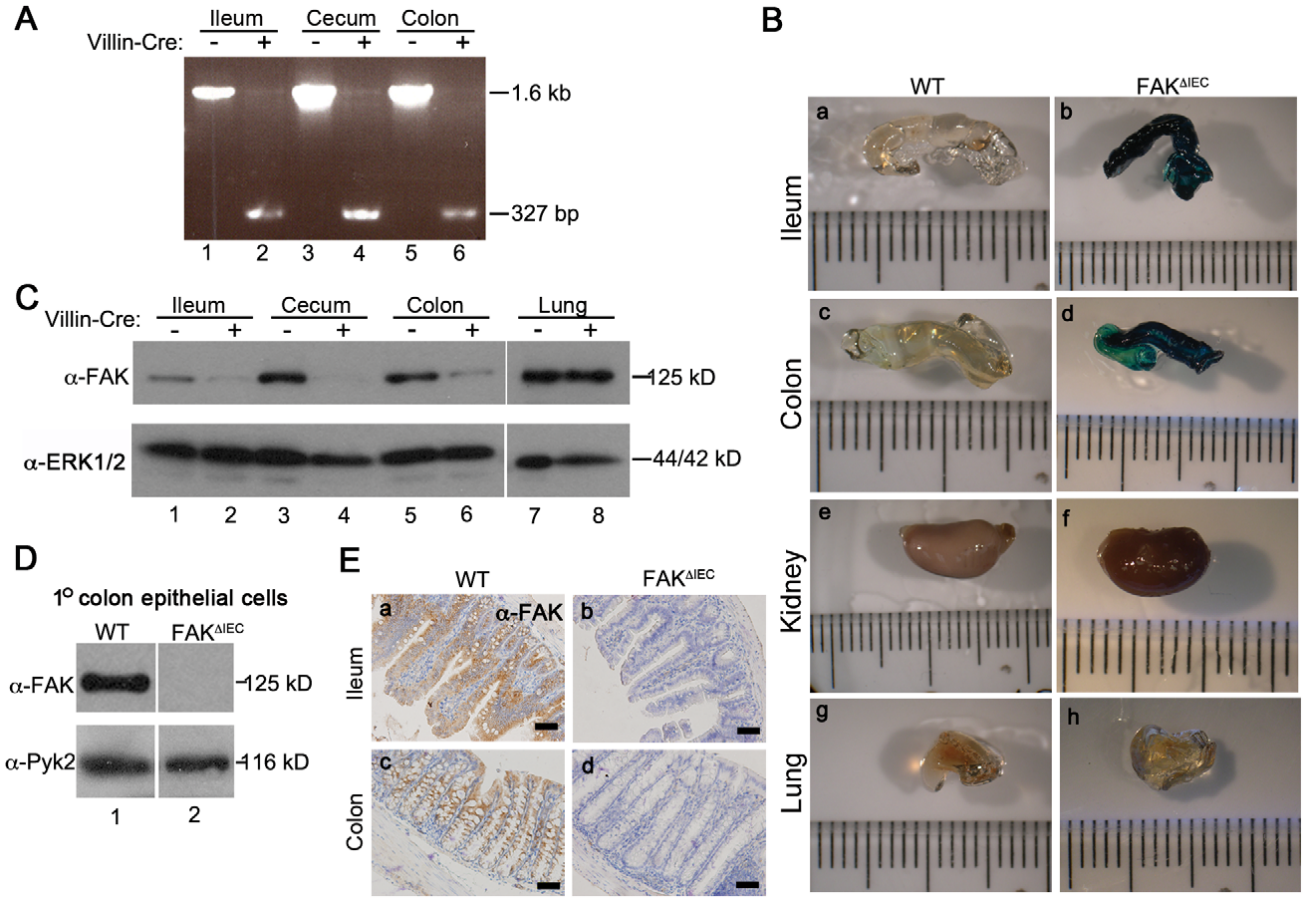
Characterization of intestinal epithelial-specific conditional FAK knockout mice. (A) PCR of DNA isolated from homogenized tissues obtained from WT and FAK^ΔIEC^ mice. The *FAK^f^* allele is 1.6 kb, the recombined locus 327 bp. (B) Whole mount X-Gal staining of tissues extracted from WT and FAK^ΔIEC^ mice. (C) Immunoblot analysis of whole organ homogenates isolated from WT and FAK^ΔIEC^ mice. The vertical line indicates non-contiguous lanes generated from a single exposure. (D) Immunoblot analysis of FAK and Pyk2 expressed in primary colonic epithelial cells. The vertical line indicates non-contiguous lanes generated from a single exposure. (E) Ileum and colon sections were immunostained for FAK. Bars represent 50 µm.

Mice harboring loxP-targeted FAK alleles (FAK^f/f^) [16] and a LacZ^f-STOP-f^ reporter allele at the ROSA26 locus [17] were crossed with mice expressing Cre recombinase under the control of the intestinal epithelial-specific villin promoter [18] (Fig. S1) to generate mice in which FAK is deleted from the entire intestinal epithelium (designated FAK^ΔIEC^). Deletion of the FAK^f^ allele in the ileum, cecum, and colon of FAK^ΔIEC^ animals was confirmed by PCR (Fig. 1A). The specificity of FAK deletion was examined by ß-galactosidase staining originating from excision of the stop codon in the ROSA26 LacZ locus (Fig. 1B). While the ileum and colon of FAK^ΔIEC^ mice stained positively for ß-galactosidase, the corresponding tissues from control (phenotypically wild type, hereafter designated WT) mice were negative. ß-galactosidase staining was negative in the kidneys and lungs of both genotypes.

Protein analysis from whole organ tissue homogenates showed greatly reduced levels of FAK in the ileum, cecum, and colon of FAK^ΔIEC^ mice compared to WT littermates (Fig. 1C). In contrast, FAK expression was normal in the lungs of FAK^ΔIEC^ mice. Primary colon epithelial cells isolated from FAK^ΔIEC^ mice contained nearly undetectable levels of FAK, whereas the related kinase Pyk2 was expressed equivalently in both WT and FAK^ΔIEC^ cells (Fig. 1D). These data were corroborated by immunohistochemistry (IHC); epithelial cells throughout the villi and crypts (including the base) of WT ileums and colons expressed FAK at steady state, while tissues derived from FAK^ΔIEC^ mice were negative (Figs. 1E and S2). This is in contrast to a recent report by Ashton *et al.* showing weak FAK expression under homeostatic conditions in the murine small intestine [19]. Consistent with our findings, however, these authors found that homeostasis in the small intestine was largely unaffected by loss of FAK, with normal villus architecture and normal numbers of both proliferating and apoptotic cells [19].

Villin is first expressed in the hindgut mesoderm at day 9 of development, and is upregulated at days 14–15 coincident with the development of intestinal villi [18]. Thus, FAK excision in villin-Cre mice is predicted to begin at embryonic day 9 and be complete by birth, making this an excellent model with which to study the role of FAK in intestinal development and homeostasis. Moreover, because villin is expressed in both the small and large intestinal epithelium, the villin-Cre model allows analysis of the entire intestinal epithelium from the proximal duodenum to the distal colon. FAK^ΔIEC^ mice were born in the expected Mendelian ratios, developed normally, and maintained body weights (Fig. S3A). Villus architecture was normal in all regions of the intestine (Figs. 1E and S3B) and expression/localization of E-cadherin and ß-catenin, major components of epithelial adherens junctions [20], were unchanged in the absence of FAK (Figs. S3C and D).

### FAK^ΔIEC^ mice are more susceptible to DSS-induced colitis

**Figure 2:**
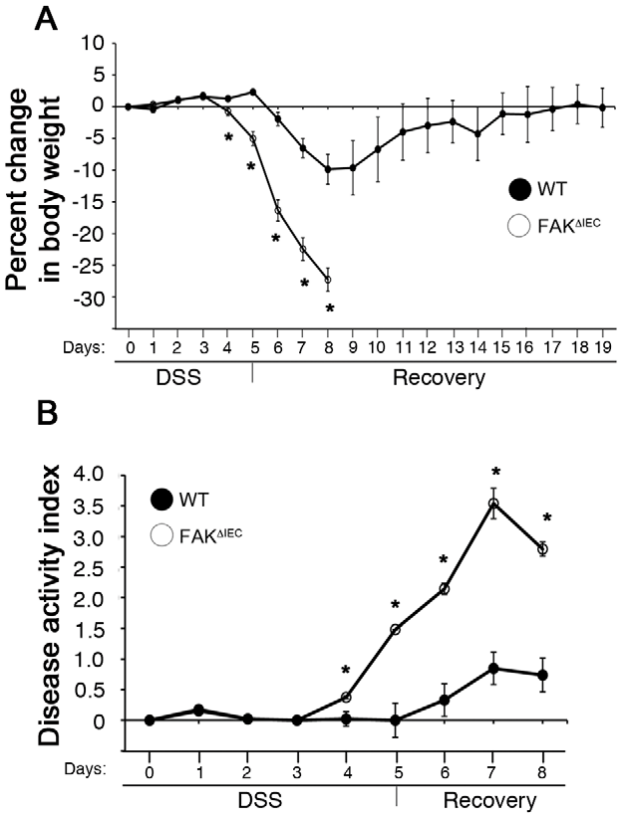
FAK^ΔIEC^ mice are more sensitive to DSS treatment. 8–12 week-old mice were fed 2.5% DSS for 5 days and allowed to recover for up to 14 days. The mean percent change in body weight (A) and disease activity index (B) are shown for 16 WT and 14 FAK^ΔIEC^ mice (days 0–5), 9 WT and 11 FAK^ΔIEC^ mice (days 6–8), and 5 WT mice (days 9–19). Asterisks indicate values that are significantly different from WT mice at the same time point (*P*<0.05).

To determine if the loss of FAK affects epithelial wound repair in the colon, we utilized an inflammatory injury model in which colitis is induced using dextran sodium sulfate (DSS) [21], [22]. Animals were given 2.5% DSS in their drinking water for 5 days, followed by a 3–14 day recovery period. As shown in Fig. 2A, WT mice began to lose weight at day 6 with a peak at day 9 (∼10% of body weight), after which they began to recover. In contrast, FAK^ΔIEC^ mice exhibited much more severe weight loss (25–30%), resulting in the need for all animals to be sacrificed by day 8. Peak levels of diarrhea and visible fecal blood were observed on day 7 in both genotypes, however the symptoms of colitis (blood in stool, loose stool consistency) were greatly aggravated in FAK^ΔIEC^ mice and correlated with a 3.5-fold higher level of disease activity compared to control animals (Fig. 2B).

**Figure 3:**
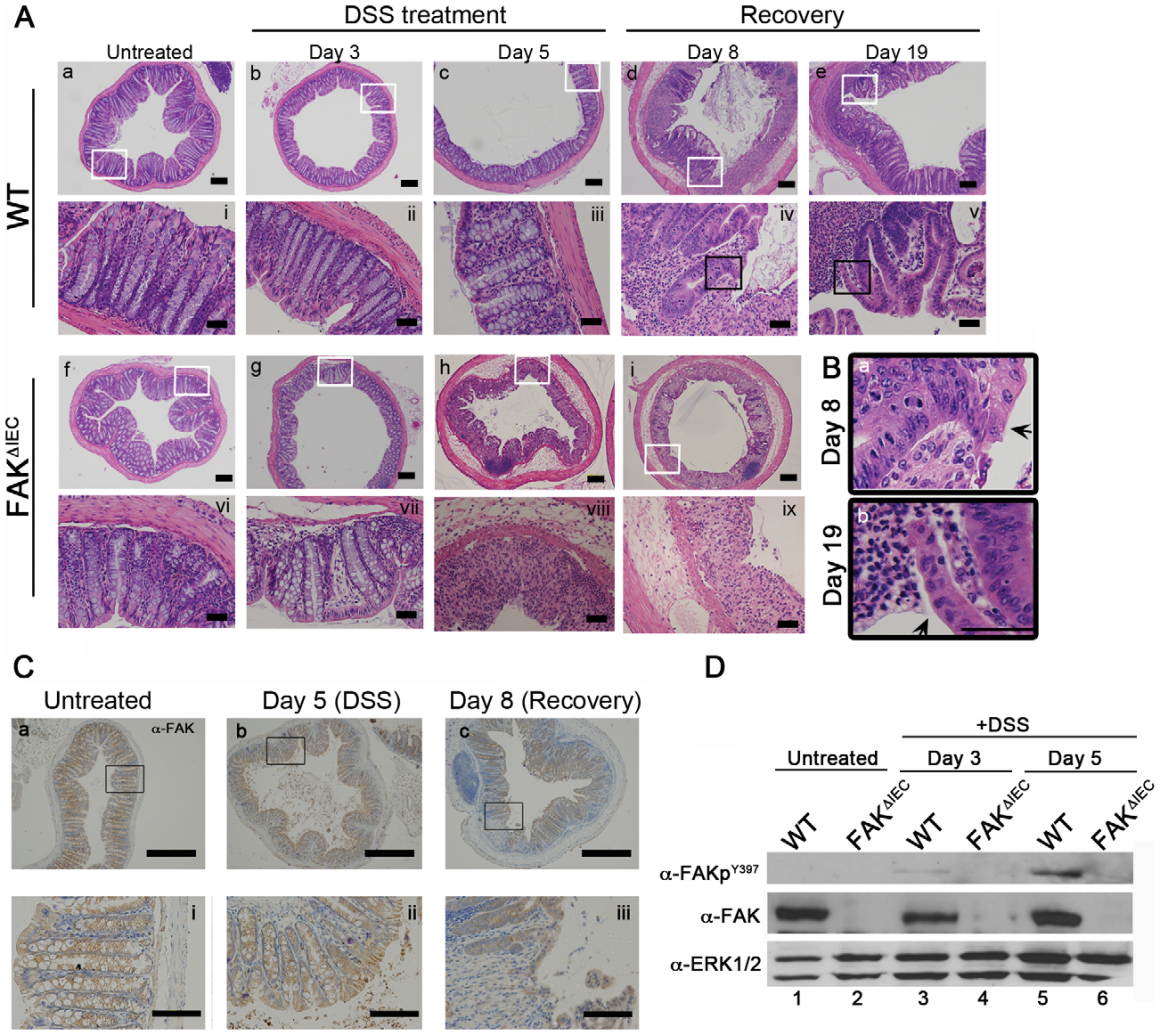
FAK^ΔIEC^ mice exhibit severe signs of epithelial erosion and edema in response to DSS treatment. (A) Representative H&E-stained colon sections from untreated and DSS-treated mice sampled at the indicated time points. Low magnification images (4×) are presented in panels a–i. Bars represent 200 µm. Higher magnification (20×) sections (white boxes) are depicted in panels i–ix. Bars represent 50 µm. (B) Regions indicated by black boxes in panels iv and v were enlarged to show detail. Arrows indicate epithelial cells overlaying ulcerated tissues. Bar represents 50 µm. (C) Colon sections from untreated or DSS-treated WT mice were immunostained for FAK at the indicated time points. Bars represent 500 µm in the low magnification (4×; panels a–c.) and 100 µm in the higher magnification (20×; panels i–iii) images. (D) Immunoblot analysis of the designated proteins expressed in primary colon epithelial cells isolated from untreated (lanes 1 and 2) or DSS-treated (lanes 3–6) WT and FAK^ΔIEC^ mice. Immunoblots are representative of 2 independent experiments containing pooled lysates from 3 animals per genotype and time point.

Damage to the colonic epithelium induced by DSS treatment is generally repaired during the recovery period [23]. At day 3 of DSS treatment, minimal changes were observed in the epithelium of both mouse genotypes (Fig. 3A, panels b, g). Tissues from WT controls remained largely intact at day 5 (panels c and iii), with patchy ulceration and edema appearing by day 8 (3 days after DSS removal; panels d and iv). Despite evidence of damage, epithelial regeneration adjacent to ulcerated areas was apparent in these mice (Fig. 3B, panel a, arrow shows epithelial cells overlaying the adjacent wound bed). By day 19, restoration of normal colonic epithelial architecture was observed in WT mice coincident with the re-emergence of crypt structures (Fig. 3A, panels e and v; Fig. 3B, panel b, arrow shows a site of re-epithelialization). In contrast to WT mice, significant tissue damage was evident in FAK^ΔIEC^ mice by day 5, characterized by pronounced edema, mucosal ulceration and loss of normal crypt structure (Fig. 3A, panels h and viii). By day 8, profound changes in FAK^ΔIEC^ colons were evident; the majority of the colonic epithelium was denuded and there was little evidence of epithelial regeneration (panels i, and high magnification panel ix). These more severe pathological responses correlated with shorter colon lengths, another indication of significant intestinal inflammation (Fig. S4). As discussed above, the FAK^ΔIEC^ mice did not survive past day 8 due to the severity of clinical symptoms.

To determine whether the morphological changes observed in response to DSS treatment coincided with increased FAK expression and/or autophosphorylation, colon sections from untreated and DSS-treated WT mice were immunostained for FAK (Fig. 3C). FAK expression remained essentially unchanged after 5 days of DSS treatment and on day 8 of the recovery period (Fig. 3C). These results were corroborated (through day 5) by immunoblotting for total FAK expression in primary colon epithelial cells (Fig. 3D, middle panel). Interestingly, FAK activity as measured by autophosphorylation at tyrosine 397 (FAKp^Y397^), was undetected in untreated animals, increased slightly by day 3 and achieved robust activation levels by day 5 of DSS treatment in control animals (Fig. 3D, upper panel). These results differ from previous studies in the small intestine, where radiation-induced injury was reported to induce a dramatic upregulation of FAK expression [19]. Here we find that, while the level of FAK protein remained unchanged, its activity was enhanced in response to mucosal injury in the colon.

### FAK expression is required for enhanced proliferation following intestinal injury

**Figure 4:**
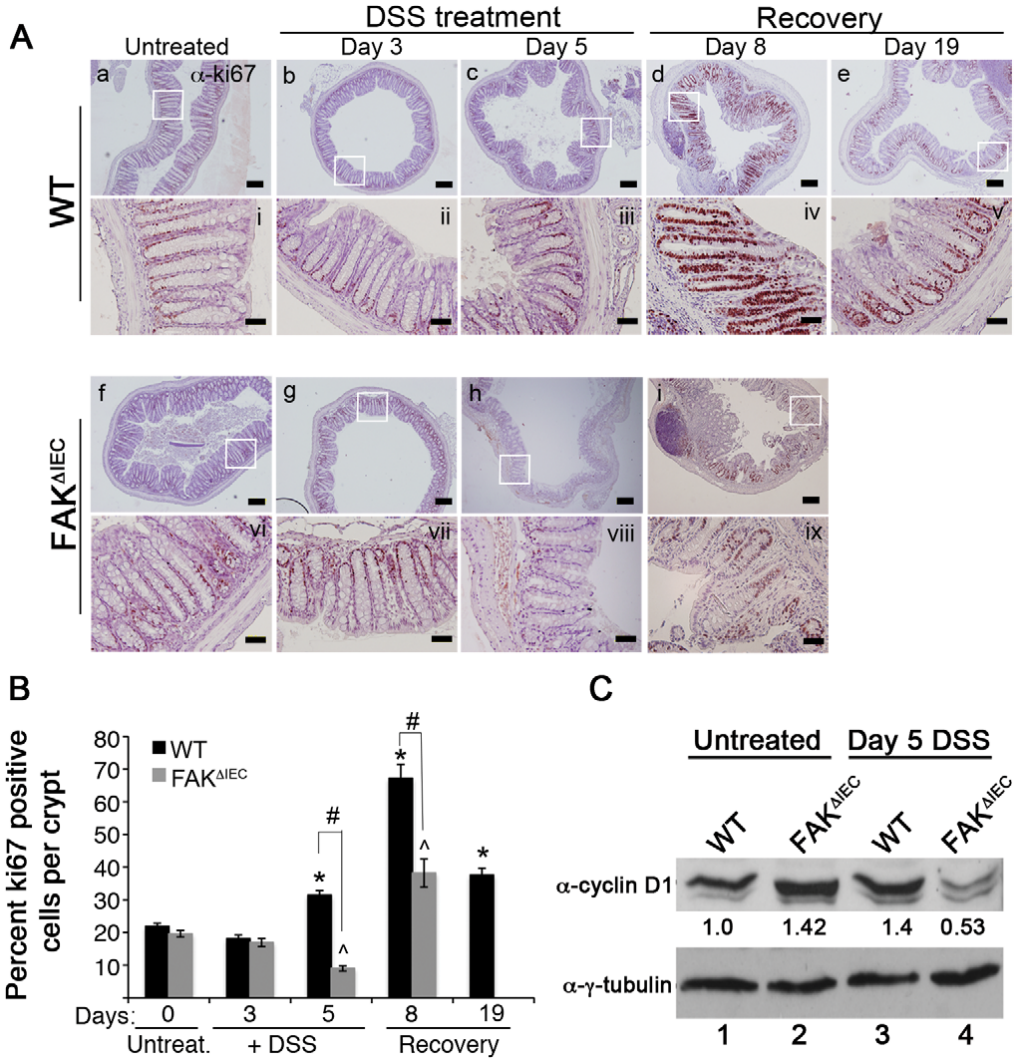
FAK controls proliferation in response to DSS treatment. (A) Colon sections from untreated and DSS-treated WT and FAK^ΔIEC^ mice were immunostained for ki67 at the indicated time points. Bars represent 200 µm in low magnification (4×; panels a–i) and 50 µm in higher magnification (20×; panels i–ix) images. (B) Percent ki67 positive cells per colonic crypt in WT and FAK^ΔIEC^ mice. Data shown are the means from 2 mice for each genotype and 40 total crypts at day 0, and 3 mice of each genotype and approximately 35–60 crypts for all other time points. Asterisks denote values that are significantly different from the mean for WT mice at day 0. ∧ indicates values that are significantly different from the mean for FAK^ΔIEC^ mice at day 0. # indicates values that are significantly different from the mean for WT mice at each time point. *P*<0.05. (D) Immunoblot analysis of the designated proteins expressed in primary colon epithelial cells isolated from untreated (lanes 1 and 2) or Day 5 DSS-treated (lanes 3 and 4) WT and FAK^ΔIEC^ mice. Total cyclin D1 levels were quantified by densitometry, normalized to the amount of tubulin present in each sample, and expressed relative to the basal level in untreated WT mice (see numbers beneath the immunoblot). Immunoblots are representative of 2 independent experiments containing pooled lysates from 3 animals per genotype and time point.

It has recently been reported that mucosal regeneration following injury depends initially on contraction of the surface epithelium around the wounded area, followed by increased epithelial cell proliferation [2]. Since FAK is known to regulate proliferation in numerous cell types [11], [24], we stained colon sections from untreated and DSS-treated mice to visualize the proliferation marker ki67. In untreated WT and FAK^ΔIEC^ mice, ki67-positive cells were restricted to the lower half of each crypt, comprising ∼18–20% of total crypt epithelial cells (Fig. 4A, panels a, i, f, and vi and Fig. 4B). A similar distribution of proliferating cells was observed through day 3 of DSS treatment, prior to signs of overt intestinal injury (panels b, ii, g, and vii). In WT animals the percentage of proliferating cells per crypt increased to 32% at day 5 and to nearly 70% at day 8 post-DSS (panels c, iii, d and iv), coincident with the epithelial regeneration seen by histological analysis (see Fig. 3A, panels d and iv). After two weeks of recovery, the percentage of ki67-positive cells per crypt in WT animals gradually decreased (Fig. 4B). In contrast, the proliferative response to DSS-induced injury was markedly attenuated in FAK^ΔIEC^ mice. The percentage of ki67-positive cells per crypt fell to 10% in these mice after 5 days of DSS treatment, and the few proliferating cells that were visible were confined to the lowest portion of each remaining crypt, adjacent to the basement membrane (Fig. 4A panels h and viii). By day 8, crypt structures were largely absent in these mice (Fig. S5); however, in those rare instances where crypts were discernable (Fig. 4A, panels i and ix), the percentage of ki67-positive cells was significantly lower than that observed in WT mice at the same time point (Fig. 4B).

### FAK-deficient epithelial cells express reduced levels of cyclin D1

Cyclin D1 is an important regulator of cyclin-dependent kinases and its expression promotes progression through the cell cycle [25], [26]. FAK has been shown to modulate cyclin D1 levels in fibroblasts, vascular smooth muscle cells, and mammary epithelial cells [11], [13]. Moreover, FAK has been shown to be required for upregulation of cyclin D1 coincident with the increased cell proliferation that occurs during injury-induced ECM remodeling [13]. To determine whether the loss of epithelial FAK modulates cyclin D1 levels, lysates isolated from colonocytes of untreated and day 5 DSS-treated animals were analyzed by immunoblot. Cyclin D1 expression was somewhat elevated in samples from untreated FAK^ΔIEC^ mice compared to WT controls (Fig. 4C, lanes 1 and 2). Following DSS treatment, cyclin D1 was seen to increase in WT colonocytes, while it underwent a significant decrease in the FAK^ΔIEC^ cells (lanes 3 and 4). This may account for the impaired proliferation seen in FAK-deficient intestinal epithelial cells after 5 days of DSS treatment.

### FAK protects intestinal epithelial cells from p53-mediated apoptosis during colitis

**Figure 5:**
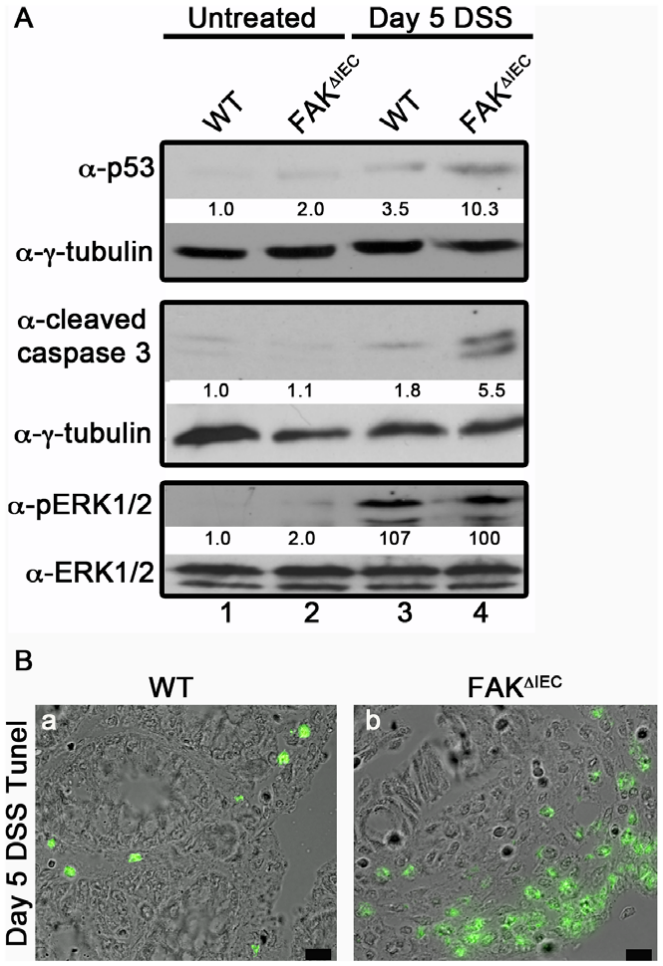
p53 accumulates in the colons of FAK-deficient mice during colitis. (A) p53, cleaved-caspase 3 and ERK1/2 present in primary colon epithelial cells isolated from untreated (lanes 1 and 2) or Day 5 DSS-treated (lanes 3 and 4) WT and FAK^ΔIEC^ mice were detected by immunoblot. Total p53, cleaved-caspase 3 and phospho-ERK1/2 levels were normalized to the amount of total tubulin (for p53 and cleaved-caspase 3) or total ERK1/2 present in each sample and expressed relative to the basal level in untreated WT mice (see numbers under each immunoblot). Immunoblots are representative of 2 independent experiments containing pooled lysates from 3 animals per genotype and time point. (B) Representative images of TUNEL-stained colon tissues from WT and FAK^ΔIEC^ mice after Day 5 DSS treatment.

In addition to its role in adhesion-mediated cell proliferation, FAK also promotes cell survival by maintaining low levels of the tumor suppressor p53. Stress signaling can activate and stabilize p53, leading to transcription of cyclin dependent kinase inhibitors, such as p21/cip1 and p27/kip1, and the induction of apoptosis [27], [28]. FAK can counteract such signaling by translocating to the cell nucleus, where it provides a scaffold that stabilizes complexes between p53 and the E3 ubiquitin ligase Mdm2, thereby causing p53 degradation [9]. Immunoblotting of epithelial cell lysates revealed that under homeostatic conditions, p53 was maintained at low levels in both WT and FAK-deficient mice (Fig. 5A, upper panels, lanes 1, 2). During DSS-induced injury, p53 levels increased 3.5-fold in colonocytes from WT animals and more than 10-fold in cells isolated from FAK^ΔIEC^ mice (lanes 3 and 4). Together these findings indicate that maintenance of low-level p53 expression at steady state does not require FAK, but that FAK acts to restrain epithelial p53 expression under conditions of inflammatory injury.

Since elevated p53 levels can also promote cell death, we investigated changes in cleaved-caspase 3 expression. Caspase 3 is an executioner caspase that, when cleaved and activated, can regulate mitochondrial events in the apoptotic pathway [29]. Similar to p53 expression, cleaved-caspase 3 levels were low in both WT and FAK^ΔIEC^ mice under steady-state conditions. (Fig. 5A, middle panels, lanes 1, 2). However, in response to DSS treatment, active caspase 3 levels increased 5.5-fold in FAK-deficient animals compared to 1.8-fold in WT mice (lanes 3, 4). Next, TUNEL-staining was performed to determine if the elevated levels of apoptotic markers observed in FAK^ΔIEC^ animals correlated with increased cell death (Fig. 5B). TUNEL-positive cells were localized to sites of tissue damage and therefore were highly prevalent in the colons of FAK-deficient mice, which exhibited greater tissue destruction in response to DSS treatment (Fig. 3A).

Finally, FAK can provide survival signals by activation of downstream signaling molecules including the mitogen-activated protein kinases (MAPK) extracellular signal-regulated kinase 1 (ERK1) and ERK2 [7]. Immunoblotting of cell lysates revealed that, as expected, ERK1/2 phosphorylation was low under homeostatic conditions, and was robustly activated after the induction of colitis. (Fig. 5A, lower panels). However, no difference in ERK1/2 phosphorylation was observed between WT and FAK^ΔIEC^ mice (lanes 3, 4). Taken together, these results show that the loss of FAK from intestinal epithelial cells results in increased p53 expression and increased sensitivity to cell death in response to DSS treatment. However, this process is uncoupled from pro-survival and/or proliferation signals generated via the ERK1/2 signaling pathway.

### Collagen deposition following mucosal injury is associated with FAK-dependent cellular proliferation in WT mice

Collagen deposition in the submucosa and mucosa is a hallmark of inflammatory bowel diseases, where it contributes to fibrosis [23], [30]. Increased ECM protein deposition and matrix crosslinking result in greater tissue stiffness [30], [31]. Interestingly, recent findings have suggested that tissue stiffness stimulates proliferation in a variety of cell types through a FAK-cyclin D1 dependent pathway [13]. Based on these data, we hypothesized that the differences in colon epithelial cell proliferation observed between WT and FAK^ΔIEC^ mice after DSS treatment could either be due to diminished collagen deposition in the absence of FAK and/or a failure of FAK^−/−^ epithelial cells to respond to proliferative cues from the ECM.

**Figure 6:**
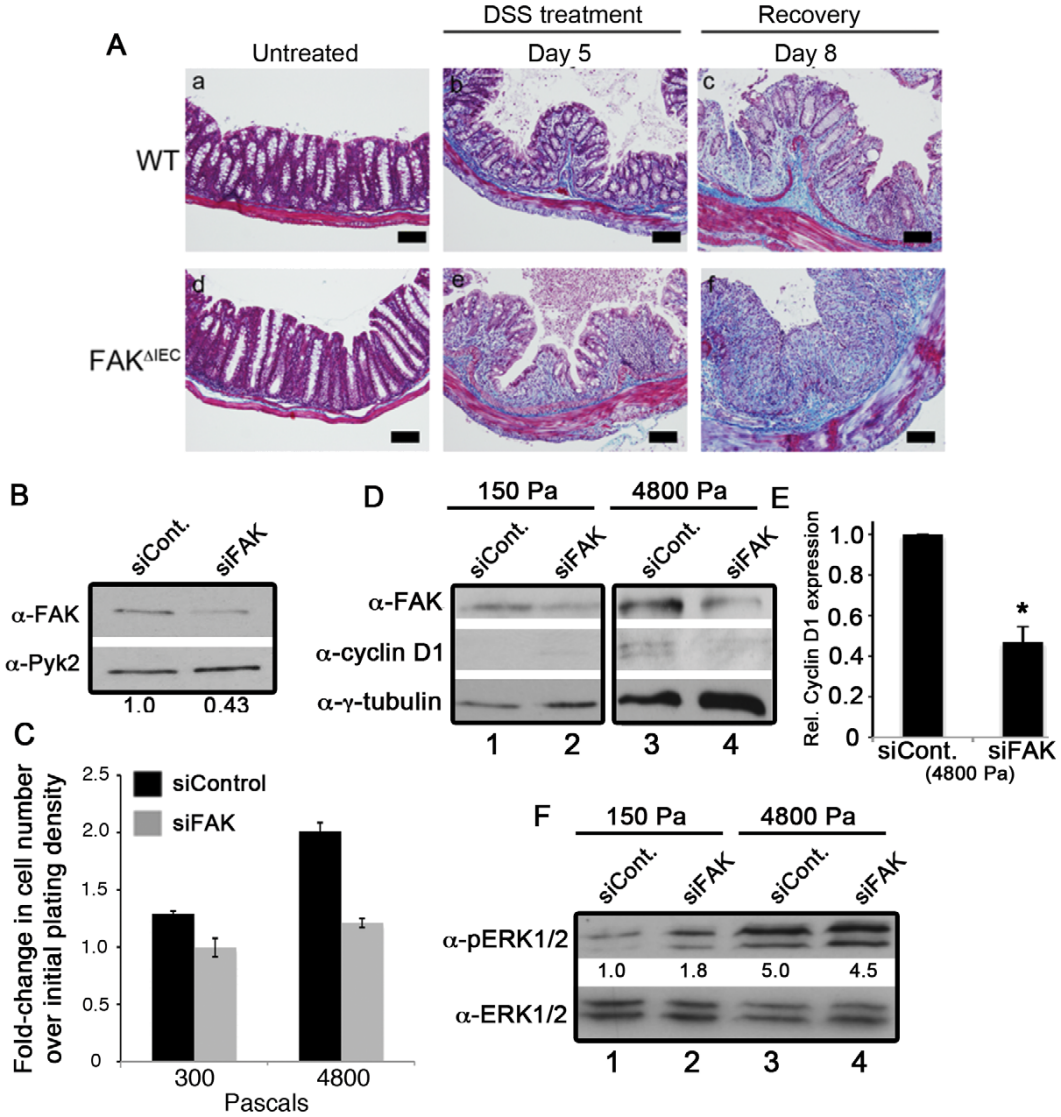
Increased tissue rigidity leads to FAK-dependent cell proliferation. (A) Colon sections from untreated and DSS-treated WT and FAK^ΔIEC^ mice were stained with Masson's trichrome stain. Collagen appears blue, muscle stains dark red, cytoplasm stains pink and nuclei appear dark brown. Bars represent 50 µm. (B) Caco-2 cells transfected with siControl or siRNA targeting FAK (siFAK) were lysed 72 hours post-siRNA transfection and immunoblotted for total FAK and Pyk2. (C) 24 hours post-transfection, cells were inoculated (6 wells per conditions) onto a soft-plate96, incubated for 48 hours, and quantified using the CyQuant NF proliferation assay. Data are representative of 2 independent experiments. (D) Caco-2 cells were transfected with siControl or siFAK for 24 hours before plating onto polyacrylamide gels with rigidities of 150 Pa or 4800 Pa, and cultured for a further 48 hours. Cells were then lysed and immunoblotted for FAK, cyclin D1 and tubulin. (E) Cyclin D1 levels were normalized to the amount of total tubulin and expressed relative to the amount of cyclin D1 in siControl-treated cells Data are representative of 3 independent experiments. Asterisks denote values that are significantly different from siControl-treated cells (*P*<0.05). (F) Caco-2 cells were transfected and plated as described in part D. Cells were then lysed and immunoblotted for phopho- and total ERK1/2. Phospho-ERK1/2 levels were normalized to total ERK1/2 and expressed relative to the amount of phospho-ERK1/2 in siControl-treated cells plated onto the 150 Pa substrate (see numbers under the immunoblot).

Collagen deposition was found to be elevated in colonic tissues from both WT and FAK^ΔIEC^ mice at days 5 and 8 following DSS treatment (Fig. 6A, see blue staining), indicating that both genotypes were capable of generating a fibrotic response to DSS-mediated colonic injury. In fact, it appears that higher levels of collagen may be deposited in FAK-deficient colons compared to WT animals following DSS treatment and this may be a consequence of the more extensive tissue damage evident in these mice. Despite elevated collagen, FAK^ΔIEC^ mice exhibited a significantly attenuated proliferative response to mucosal injury. To investigate whether FAK directly regulates the proliferative response of intestinal epithelial cells to tissue rigidity, Caco-2 intestinal epithelial cells were depleted of endogenous FAK by siRNA, then plated for 2 days on collagen-coated polyacrylamide substrates of rigidities ranging from 300 Pa (similar to the rigidity of lung tissue) to 4800 Pa (similar to the rigidity of muscle cells) [31]. FAK expression in siRNA-treated cells was reduced by approximately 60% while Pyk2 levels remained unaffected (Fig. 6B). Plating efficiency and adhesion of cells to the different substrates was identical between siControl and siFAK treated cells (data not shown). After 48 hours, the number of control cells present on the low rigidity substrate (300 Pa) increased slightly from the initial plating density (∼1.3 fold; Fig. 6C). Control cells plated on the higher rigidity substrate (4800 Pa) showed a larger net gain (2-fold), indicative of greater survival/proliferation rates on the more rigid substrate. In contrast, FAK-depleted cells showed no significant net increase during this 48-hour period on either of the matrix rigidities tested. These data demonstrate that FAK regulates the proliferative response to matrix rigidity in these intestinal epithelial cells.

Since the loss of FAK from intestinal epithelial cells *in vivo* resulted in less proliferation coincident with reduced cyclin D1 expression after DSS treatment (Fig. 4), we next tested whether FAK depletion in Caco-2 cells affected cyclin D1 levels when plated onto substrates of high and low rigidity. Cyclin D1 was not detected in either siControl- or siFAK-treated cells plated on the low rigidity substrate (Fig. 6D, middle panel, lanes 1, 2), further supporting our findings that Caco-2 cells fail to proliferate on soft matrix even in the presence of FAK. Cyclin D1 was present in siControl-treated cells cultured on the higher rigidity substrate (4800 Pa; lane 3); however, we were unable to detect an increase in FAK autophosphorylation under these conditions (data not shown). In comparison, cyclin D1 levels were significantly lower in the siFAK-treated cells (lane 4 and Fig. 6E), consistent with a failure of these cells to proliferate under these conditions. Finally, immunoblotting of siControl and siFAK-treated lysates revealed that ERK1/2 phosphorylation was lower in cells plated onto soft matrix, and was robustly activated after plating onto the high rigidity substrate (Fig. 6F). Similar to our *in vivo* findings after DSS treatment (Fig. 5A), no difference in ERK1/2 phosphorylation was observed between control-treated or FAK-depleted cells (lanes 3, 4).

## Discussion

### FAK is not required for normal intestinal development and homeostasis

FAK has an established role in many cellular processes involved in intestinal homeostasis, including cell proliferation, survival, and migration [32]. Despite these functions, our studies show that deletion of FAK in the intestinal epithelial cell lineage early during development has no significant consequence on the architecture of the small or large intestine under homeostatic conditions. A similar result was reported when FAK was acutely deleted from small intestinal crypts in adult animals; epithelial cell proliferation, migration, differentiation, and survival all appeared normal within this tissue under homeostatic conditions [19]. FAK is thought to regulate these diverse cellular functions largely through its role in adhesion signaling downstream of integrins, including ß1, ß3 and ß5 integrin receptors [33], [34], [35]. Thus it is interesting that conditional loss of FAK does not phenocopy loss of ß1 integrin expression in intestinal crypts [36]. Using the same villin-Cre transgenic model as we employed here, intestinal crypts lacking ß1 integrin exhibited hyperplasia and underwent aberrant enterocyte differentiation in the absence of any environmental insult [36], resulting in postnatal death between days 7 and 14 due to malnutrition. One possible explanation for these differences is functional redundancy between FAK and its only other family member Pyk2, which we found is also expressed in the intestinal epithelium (Fig. 1D). Pyk2 has been shown to control p53 levels, cell cycle progression, and proliferation in ovarian carcinoma cells that express both FAK and Pyk2, and in FAK^−/−^ mouse embryo fibroblasts that undergo upregulation of Pyk2 due to loss of FAK [37]. Members of the Src family of kinases (SFKs) also perform multiple functions in the adhesion-mediated control of proliferation, adhesion, spreading and migration [3], [34], [38]. Indeed, SFKs have been shown previously to suppress apoptotic signaling in both human and rodent enterocyte cell lines [4], [39], [40], [41]. Based on these data, we suggest that the absence of any clear phenotype exhibited by the FAK^ΔIEC^ mice during development and under homeostatic conditions may be due to proliferation and survival signals emanating from Pyk2 and/or other transducers of integrin signaling.

### FAK promotes cell survival and regulates the proliferative response to intestinal epithelial injury

While the loss of FAK had no apparent effect on small or large intestinal architecture or function under homeostatic conditions, it had a profound outcome on the response to epithelial injury. Indeed, our data indicate that FAK serves an essential role in colonic epithelial regeneration by contributing to epithelial cell survival and proliferation under conditions of mucosal injury. In response to cellular stress, accumulation of the tumor suppressor protein p53 stimulates the transcription of a number genes promoting growth arrest and/or cell death. We found that FAK activity increased in the colonic epithelium of WT mice following DSS treatment and, while p53 levels also rose, they did not do so to the same extent as in FAK^ΔIEC^ mice. Similarly, while colonocytes from WT animals exhibited a slight increase in activated-caspase 3 during colitis, this level rose dramatically in FAK-deficient mice and was coincident with increased numbers of TUNEL-positive cells. These data suggest that canonical FAK signal transduction pathways are activated following DSS-induced damage, which then promote cell survival by preventing an increase in expression of p53 and other pro-apoptotic molecules. These results are consistent with findings by Lim *et al.* [9], which show FAK facilitates cell survival through enhanced p53 degradation under conditions of cellular stress. However, our observations differ somewhat from those reported by Ashton *et al.*, who showed that FAK expression (but not activity) was elevated in the small intestine in response to gamma irradiation [19]. These authors also reported that p53 expression remained unchanged in response to DNA damage in FAK-deficient enterocytes [19]. These differences may reflect distinctions between injury models or fundamental differences in the outcome of FAK-dependent signaling between the small intestine and the colon.

In addition to higher than normal levels of p53, the reduced epithelial proliferation observed in FAK-deficient mice also coincided with significantly diminished cyclin D1 expression following inflammatory injury (Fig. 4). Several reports link FAK expression to the induction of cyclin D1 in cultured cells [11], [12]. Indeed, FAK promotes cyclin D1 transcription by stimulating the expression of two transcription factors, an Ets-like transcription factor and Kruppel-like factor 8 [11], [12]. Conversely, cyclin D1 mRNA expression is suppressed by expression of either FAK^Y397F^ or FAK-related non kinase (FRNK, the non-catalytic carboxy terminal domain of FAK) [13]. Interestingly, we found that ERK activation in response to DSS treatment was independent of FAK. Similarly, ERK phosphorylation occurred in FAK-depleted Caco-2 cells plated onto higher rigidity substrate. It has been reported previously that physical forces such as cyclic deformation induce Caco-2 cell proliferation in a FAK-ERK dependent manner [42]. However, our observations are consistent with findings by Klein *et al.* indicating that the regulation of mitogenesis and cyclin D1 expression by extracellular matrix stiffness requires FAK rather than ERK1/2. These results highlight the complex nature of the cellular response to colonic injury and the fact that the requirement for FAK during this process appears to be both cell type- and context-specific.

### FAK functions as a mechanosensor to control intestinal epithelial proliferation

A hallmark of inflammatory injury is the deposition of collagen matrix within the inflamed tissue [15], [23], [30], [43], [44]. Elevated collagen expression in the mucosa and submucosa of DSS-treated animals induces fibrotic thickening and contributes to increased rigidity within colonic tissues [23]. We found that collagen deposition following DSS treatment in WT animals coincided temporally with a marked increase in colonic epithelial cell proliferation and elevated FAK activity. In addition to increased stiffening, it is also possible that FAK activation occurs in direct response to elevated levels of collagen and other ECM components, such as fibronectin. Arterial stiffening due to vascular injury promotes a similar proliferative response to damage [13], and greater collagen matrix density in the mammary epithelium increases tissue rigidity and promotes cellular proliferation and tumorigenesis in a FAK-dependent manner [45]. These data are consistent with our findings, in that FAK^ΔIEC^ mice showed decreased cyclin D1 expression and an attenuated proliferative response to inflammatory injury despite robust collagen deposition in the injured colon.

**Figure 7:**
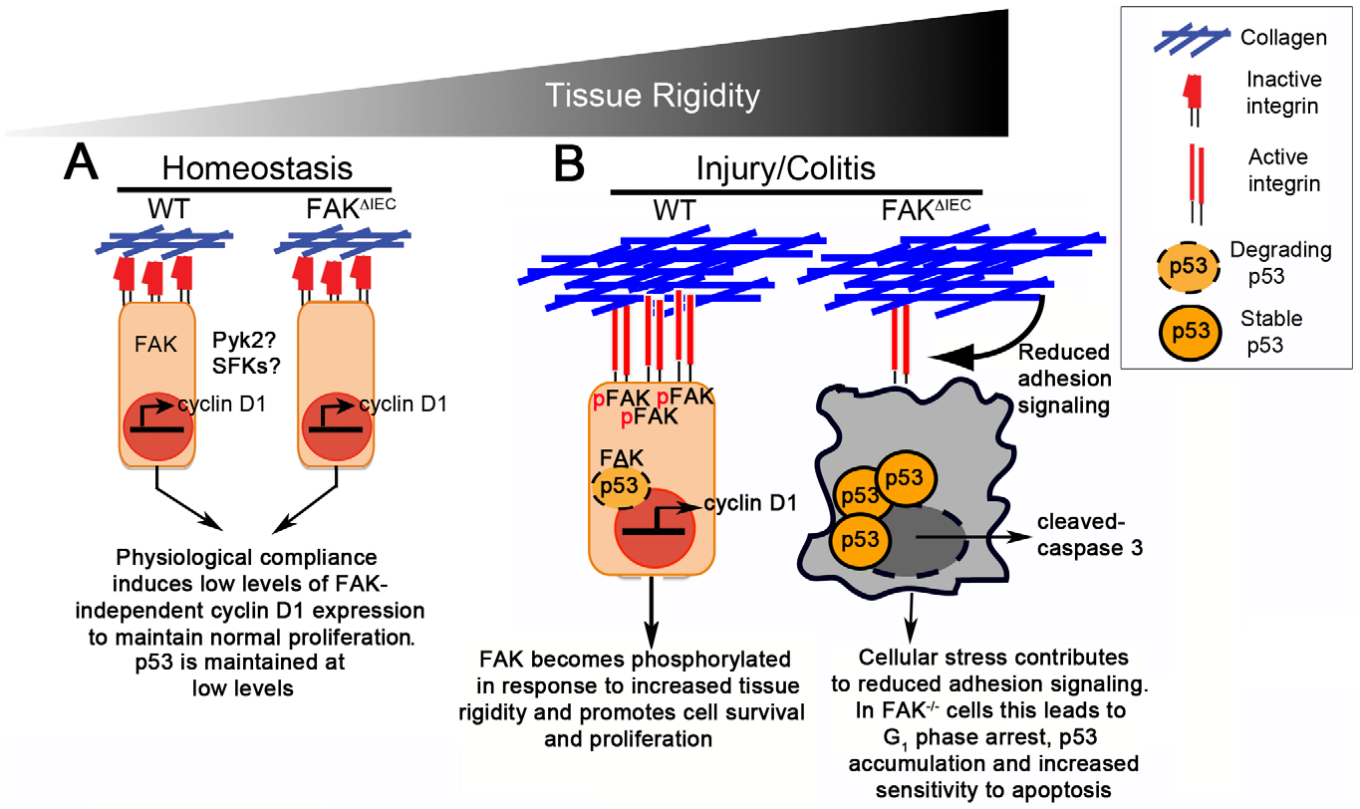
Increased tissue rigidity drives cell cycle progression in a FAK-cyclin D1-dependent manner. (A) Under homeostatic or physiological conditions, where tissue compliance is relatively high, FAK and integrin receptors are minimally associated and cyclin D1/proliferation are kept to basal levels. p53 levels are also maintained at low levels. (B) Induction of colitis leads to the deposition of collagen and other ECM components. The resultant increased tissue rigidity promotes FAK-integrin complexes, which in turn induces FAK phosphorylation and promotes cell survival. FAK auto-phosphorylation can result in Akt activation and subsequent phosphorylation of Mdm2. FAK translocation to the nucleus also allows FAK to function as a scaffold, stabilizing p53-Mdm2 complexes. Both of these FAK signaling pathways enhance cell survival by keeping p53 levels low. Finally, FAK contributes to the induction of cyclin D1 expression by upregulating transcription factors such as krupple-like factor 8 (KLF-8). In the absence of FAK, the failure to increase cyclin D1 levels significantly attenuates proliferation and impairs the healing response.

Findings by Klein *et al.* suggest that cells respond to increasing extracellular stiffness by regulating the degree to which FAK stably associates with activated integrins independent of its kinase activity [13]. This is highlighted by the fact that cells expressing constitutively active FAK are unable to progress through the cell cycle on low stiffness matrices [13], and is supported by our data showing that, in the absence of damage and subsequent tissue stiffening, FAK is not required to maintain homeostasis in the colon (see model, Fig. 7A). During colitis, however, we hypothesize that pathological changes in matrix elasticity within the colon promote formation of FAK-integrin complexes, cyclin D1 upregulation and progression through the cell cycle (Fig. 7B). Within this context, FAK also becomes activated and mediates cell survival by maintaining low levels of pro-apoptotic molecules like p53 and activated-caspase 3. In the absence of FAK, loss of adhesion signaling leads to a reduction in cyclin D1 levels, inhibited proliferation and an accumulation in p53 expression. This is further supported by our *in vitro* data, which showed impaired proliferation of FAK-depleted Caco-2 colonic epithelial cells on more rigid substrates coincident with reduced levels of cyclin D1.

The pressure to maintain the integrity of the epithelial barrier is paramount to host survival. Re-epithelialization is therefore an essential component of the healing process following gastrointestinal damage associated with inflammatory bowel diseases. Under conditions of stress, such as that induced by prolonged exposure to inflammatory mediators, we suggest that FAK tips the balance in favor of cell survival while coordinately promoting an appropriate proliferative response required to regenerate damaged mucosal surfaces. The current study also supports a potential mechanism implicating FAK as a regulator of intestinal epithelial cell proliferation through the mechanotransduction of signals emanating from the tissue matrix. By establishing the mechanisms through which epithelial repair is regulated, it may be possible to develop better treatment options for inflammatory bowel diseases.

## Materials and Methods

### Ethics statement

The animal studies were carried out in strict accordance with the recommendations in the Guide for the Care and Use of Laboratory Animals of the National Institutes of Health. The protocol was approved by the University of Virginia Animals Care and Use Committee (Protocol Number 3158). All efforts were made to keep pain and suffering to a minimum.

### Intestinal-specific conditional FAK knockout mice

Mice were generated as described in supplemental materials to produce Villin^wt/Cre^FAK^f/f^ROSA26LacZ^f-STOP-f/f-STOP-f^ (designated FAK^ΔIEC^) and Villin^wt/wt^FAK^f/f^ROSA26LacZ^f-STOP-f/f-STOP-f^ (designated WT) littermate controls. All mice are in a pure C57BL/6 background. Animal experiments were approved by the institutional animal care and use committee of UVA.

**Genotyping of mice and analysis of Cre-mediated recombination** (see Methods S1)

### Antibodies and reagents

Immunoblot and immunohistochemical analyses were performed using the following antibodies: polyclonal phospho-FAK^pY397^ (BD Transduction Laboratories, San Jose, CA), polyclonal anti-ERK1/2, polyclonal phospho-ERK1/2 and monoclonal cleaved-cleaved caspase 3 were all from Cell Signaling (Danvers, MA), polyclonal anti-FAK C-20, monoclonal anti-p53 and monoclonal anti-FAK A-17 were all from Santa Cruz Biotechnology, Inc, (Santa Cruz, CA). Monoclonal anti-ki67 clone TEC-3 (DakoCytomation, Denmark), monoclonal anti-E-cadherin (BD Transduction Laboratories), monoclonal anti-ß-catenin (Epitomics, Burlingame CA), monoclonal anti-Pyk2 (BD Transduction Laboratories), polyclonal anti-cyclin D1 (Abcam, Cambridge, MA) and monoclonal anti-gamma-tubulin (Sigma-Aldrich, St. Louis, MO) were all purchased from the suppliers indicated.

### siRNA transfection

20 µM siRNA oligonucleotides [46] targeting human FAK (Dharmacon, Lafayette, CO) and non-targeting controls (siControl, Ambion, Austin, TX) were transfected using Lipofectamine RNAi max (Invitrogen, Carlsbad, CA) according to manufacturer's specifications.

### ß-galactosidase staining

Organs were rinsed in cold phosphate-buffered saline (PBS), fixed in 0.1 M sodium phosphate, 20 mM Tris pH 7.3, 5 mM EGTA, 2 mM magnesium chloride, 0.25% glutaraldehyde, 1% formaldehyde for 30 minutes, and stained overnight at 4°C in 0.1 M sodium phosphate, 20 mM Tris pH 7.3, 2 mM magnesium chloride, 5 mM potassium ferrocyanate, 5 mM potassium ferricyanate, 0.1% deoxycholate, 0.2% NP40, 1 mg/mL 5-bromo-4-chloro-3-indolyl-ß-D-galactopyranoside (X-Gal). Organs were then washed in PBS, dehydrated in methanol, and placed in 2∶1 benzylbenzoate∶benzyl alcohol for 48 hours prior to visualization.

### DSS treatment

8–12 week old mice were given 2.5% dextran sulfate sodium (DSS; MP Biomedicals, LLC, Solon, OH) in their drinking water for 5 days and allowed to recover for up to 14 days. Disease activity index (DAI) was calculated based on change in body weight, presence of blood in the stool, and stool consistency, as previously described [47]. The scores were determined as follows: change in weight (0:<1%, 1: 1–5%, 2: 6–10%, 3: 11–15%, 4: >15%), stool consistency (0: normal, 2: loose stools, 4: diarrhea), and stool blood (0: negative, 2: positive, 4: gross bleeding). The total score was then divided by 3. Following observation, colons were excised, measured, and processed for analysis at the indicated time points.

### Immunostaining

Intestinal tissues were flushed with PBS and fixed overnight in Bouin's fixative (Ricca Chemical Company, Arlington, TX), 10% formalin or snap-frozen in O.C.T. (Sakura Finetek U.S.A., Inc., Torrance, CA). Tissues were subsequently stained for H&E, immunohistochemistry, or immunofluorescence (see Methods S2). For the detection of apoptotic cells, TUNEL staining was performed as per the manufacturer's instructions (Roche, Indianapolis, IN).

### Protein isolation from intestinal epithelial cells

The ileum and colon were opened longitudinally, washed extensively with Hank's Buffered Salt Solution (HBSS), cut into 3–5 mm pieces, and incubated on an orbital shaker in HBSS, 5% FBS, 2 mM EDTA at 37°C for 20 minutes. The supernatants were collected, filtered through a 100 µm filter, and spun at 4°C for 10 minutes at 1800 rpm. Pellets were washed, lysed, and analyzed by immunoblotting [48], [49]. For some experiments, colon sections were flushed with ice-cold PBS containing protease (1 mM PMSF, 0.15 U/ml aprotinin and 1 µg/ml each of leupeptin, pepstatin and antipain) and phosphatase inhibitors (1 mM EDTA, 1 mM NaF, 20 mM Na_4_P_2_0_7_ and 2 mM Na_3_VO_4_). Colons were opened longitudinally and scraped to isolate mucosal protein. Scrapped cells were placed in cold cell extraction buffer (10 mM Tris, 100 mM NaCl, 1 mM EGTA, 1% Triton X-100, 10% glycerol, 0.1% SDS, 0.5% deoxycholate supplemented with protease and phosphatase inhibitors at the concentrations listed above). Mucosal scrapings were briefly homogenized with a Tissue Master 240 (Omni International, Kennesaw, GA) on ice before centrifugation at 4°C for 10 minutes at 13,000 rpm to remove cell membranes and debris. Lysates were then analyzed by immunoblotting. To quantify changes in protein expression levels densitometry was performed. Band intensities were quantified using ImageJ (National Institutes of Health).

### Soft-plate96 proliferation assays and polyacrylamide substrates

Multiwell “soft-plates” were inoculated for cell growth assays on substrates of increasing rigidity as described in Tilghman *et al.* [50] (see Methods S3). Flexible polyacrylamide substrates were generated on glass coverslips and adapted for cell culture using the method of Pelham and Wang and as described in Tighman *et al.*

### Statistical methods

A two-sample t-test, assuming unequal variance, was used to determine statistical significance between conditions.

## Supporting Information

Figure S1 **Cre-mediated recombination in FAK^ΔIEC^ mice.** Schematic diagram of the *FAK* ^f^ and *ROSA26LacZ* ^f-STOP-f^ loci following Villin-driven Cre-mediated recombination. The second kinase domain exon of FAK (black box) is flanked by loxP sites (black triangles). A stop codon on the ROSA26 locus is also flanked by loxP sites (black triangles). Primers (short arrows) and PCR products (thin lines) are shown for each allele.
(TIF)

Figure S2 **Efficient FAK deletion occurs at the base of intestinal crypts in FAK^ΔIEC^ mice.** Ileum and colon sections from WT and FAK^ΔIEC^ mice were immunostained for FAK. Panels represent an enlarged region from crypts of ileum (a, b) and colon (c, d). Images show positive FAK staining in the base of crypts from WT animals that is absent in crypts from FAK^ΔIEC^ animals. Bar represents 50 µm.
(TIF)

Figure S3 **IEC-specific conditional FAK knockout mice maintain normal weight patterns and gut architecture compared to controls.** (A) Average body weight in grams of 8–10 week-old WT and FAK^ΔIEC^ mice. Data presented are the average of 12 WT and 11 FAK^ΔIEC^ mice. (B) H&E stained ileum and colon sections isolated from 8–10 week-old WT and FAK^ΔIEC^ mice. (C) Immunoblot analysis of E-cadherin and ß-catenin protein present in primary colonocytes isolated from WT and FAK^ΔIEC^ mice. The vertical line separator is indicative of non-contiguous lanes on the gel. However, immunoblots shown for each antibody were generated from a single exposure. (D) Colon sections from WT and FAK^ΔIEC^ mice were stained for E-cadherin (green staining) and examined by immunofluorescence. Bars represent 10 µm (panels a, b). Panels c and d represent enlarged regions from panel a and b respectively. Arrows depict regions of membrane-associated E-cadherin staining.
(TIF)

Figure S4 **DSS-induced colonic shortening is aggravated in FAK^ΔIEC^ mice.** Colon length measured in centimeters from untreated and DSS-treated WT and FAK^ΔIEC^ mice. Asterisks indicate values that are significantly different from untreated WT mice (Day 0). ∧ indicate values that are significantly different from WT mice at the same time point. In both cases, p<0.05.
(TIF)

Figure S5 **Colons of FAK^ΔIEC^ mice generally lack crypt structure at day 8 post-DSS treatment.** Low magnification images of ki67-stained DSS-treated colon sections from WT (panel a) and FAK^ΔIEC^ (panel b) animals (day 8). Bars represents 200 µm. Panels c and d show high magnification images of panels a and b, respectively. Bars represents 50 µm.
(TIF)

Methods S1 **Genotyping of mice and analysis of Cre-mediated recombination.** Animals were genotyped using tail DNA and subjected to PCR analysis. The following primers were used for PCR of the FAK locus: P1 (5′-GAGAATCCAGCTTTGGCTGTTG-3′) and GenoRV (5′-GAATGCTACAGGAACCAAATAAC-3′). This primer set generates 290-bp (WT) and 400-bp (FAK^f^) products. To determine the status of the villin locus, the following primers were used: MR1878 (5′- GTGTGGGACAGAGAACAAACC-3′) and MR1879 (5′- ACATCTTCAGGTTCTGCGGG-3′). These primers generate an 1100-bp (Villin-Cre) product. For PCR of the ROSA26 allele, the following primers were used: ROSA1 (5′- AAAGTCGCTCTGAGTTGTTAT-3′), ROSA2: (5′-GCGAAGAGTTTGTCCTCAACC-3′) and ROSA3: (5′- GGAGCGGGAGAAATGGATATG-3′). ROSA1 and ROSA3 primer sets generate a 600-bp product containing the WT allele, while ROSA1 and ROSA2 primer sets generate a 300-bp product containing the ROSA26LacZ^f-STOP-f^ allele. To check for Cre-mediated recombination in intestinal tissues, DNA was isolated from homogenates of intestinal tissues (ileum, cecum, colon) and subjected to PCR. The following primers were used: LoxP (5′GACCTTCAACTTCTCATTTCTCCC-3′) and GenoRV (see above). These primers amplified products consisting of a *FAK^f/f^* (1.6 kb), and a Cre-mediated recombined fragment (327 bp). PCR fragments were separated on 1.5% agarose gels.
(DOCX)

Methods S2 **Preparation of tissue sections and immunohistochemical staining.** Ileum and colon tissues were flushed with PBS to remove fecal material. Tissues were then fixed overnight in Bouin's fixative (Ricca Chemical Company, Arlington, TX), 10% formalin or snap-frozen in O.C.T. (optimal cutting temperature, Sakura Finetek U.S.A., Inc., Torrance, CA). Following fixation, tissues were washed in 70% ethanol. Segments were then embedded in paraffin directly or cut and mounted in agar-10% formalin prior to being embedded in paraffin. Five-micron paraffin sections were cut and mounted onto slides. For immunohistochemical (IHC) staining, slides were deparaffinized in a series of xylene and ethanol baths. Slides were treated in 0.3% hydrogen peroxide to block endogenous peroxidase activity. Antigen retrieval was performed by microwaving slides for 20 minutes in 10 mM sodium citrate buffer (pH 6.0). IHC staining was performed utilizing a biotin blocking kit and Vectastain ELITE ABC kit as per manufacturer recommendations (Vector Laboratories, Burlingame, CA). Slides were incubated with primary antibodies in PBS containing Vector blocking agent. Biotinylated secondary anti-rat, anti-rabbit, or anti-mouse antibodies (Vector Labs) were added and incubated for 10 minutes at room temperature. Sections were then incubated with Nova Red or 3,3-Diaminobenzidine (DAB) substrate (Vector Labs) followed by a hematoxylin counterstain. For analysis of tissue architecture, hematoxylin and eosin (H & E) staining was performed. To visualize connective tissue, Masson's trichrome staining was performed. For immunofluorescence, five-micron frozen sections were cut and mounted onto slides. Sections were blocked and stained in 10% goat serum at room temperature. Sections were incubated with primary antibodies for 1 hour followed by a 30 minute incubation with anti-mouse fluorescein isothyocyanate (FITC)-conjugated secondary antibodies (Jackson ImmunoResearch, West Grove, PA). Cells were imaged with a Nikon Eclipse E800 microscope connected to a charged-coupled device (CCD) camera. Imaging was performed using Openlab software (Perkin Elmer, Waltham, MA, USA). IHC- and H & E-stained sections were examined with an Olympus BX51 microscope and images were acquired with an Olympus DP70 digital camera controlled by Image Pro Plus™ software (EPIX, Inc, Buffalo Grove, IL).
(DOCX)

Methods S3 **Soft-plate96 proliferation assays.** To assay cell growth on substrates of increasing rigidity, we employed the use of a multiwell “soft-plate” as described in Tilghman *et al.* [49]. Soft-plate96 assay plates were seeded with siControl or siFAK-treated cells in sextuplet wells 24 hours post-siRNA transfection at a density of 5000 cells per well, and the cells were allowed to proliferate for a further 48 hours. Cell proliferation was measured using the CyQuant NF cell proliferation assay kit (Invitrogen, Carlsbad, CA). Standard curves were generated for each experiment by performing serial dilutions of the cells in an empty row of wells and allowing them to adhere for four to six hours prior to quantification with CyQuant.
(DOCX)
