# Supplementary figures and images for: FAK Regulates Intestinal Epithelial Cell Survival and Proliferation during Mucosal Wound Healing
Source: PLoS One. 2011 Aug 24;6(8):e23123. doi: 10.1371/journal.pone.0023123 (PMC3160839; doi:10.1371/journal.pone.0023123)

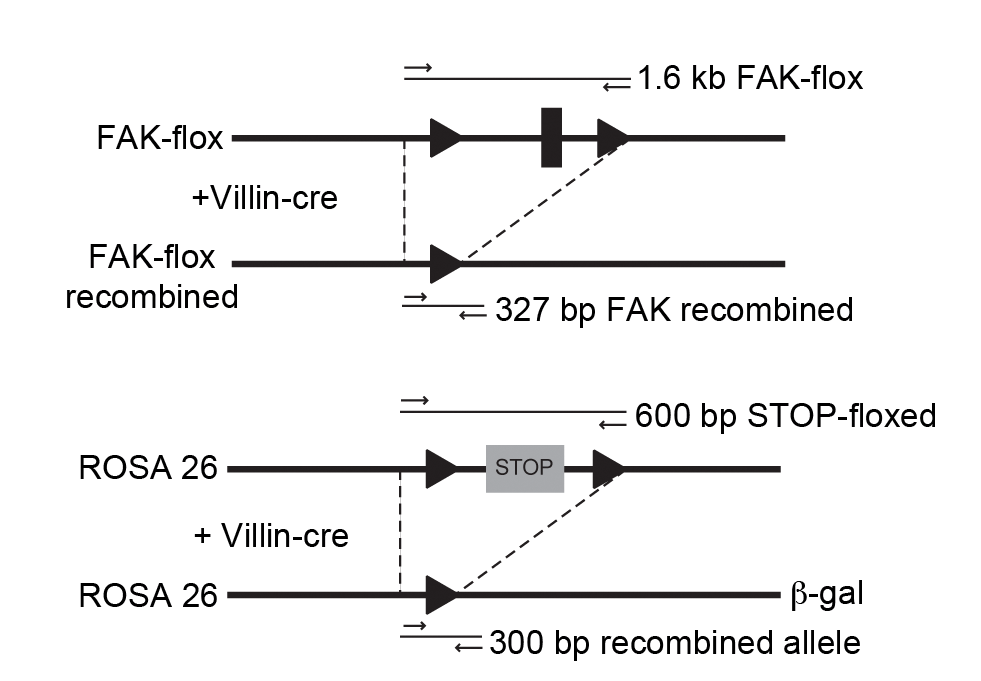

Supplement: Figure S1 — Cre-mediated recombination in FAKΔIEC mice. Schematic diagram of the FAK f and ROSA26LacZ f-STOP-f loci following Villin-driven Cre-mediated recombination. The second kinase domain exon of FAK (black box) is flanked by loxP sites (black triangles). A stop codon on the ROSA26 locus is also flanked by loxP sites (black triangles). Primers (short arrows) and PCR products (thin lines) are shown for each allele. (TIF) [file pone.0023123.s001.tif]

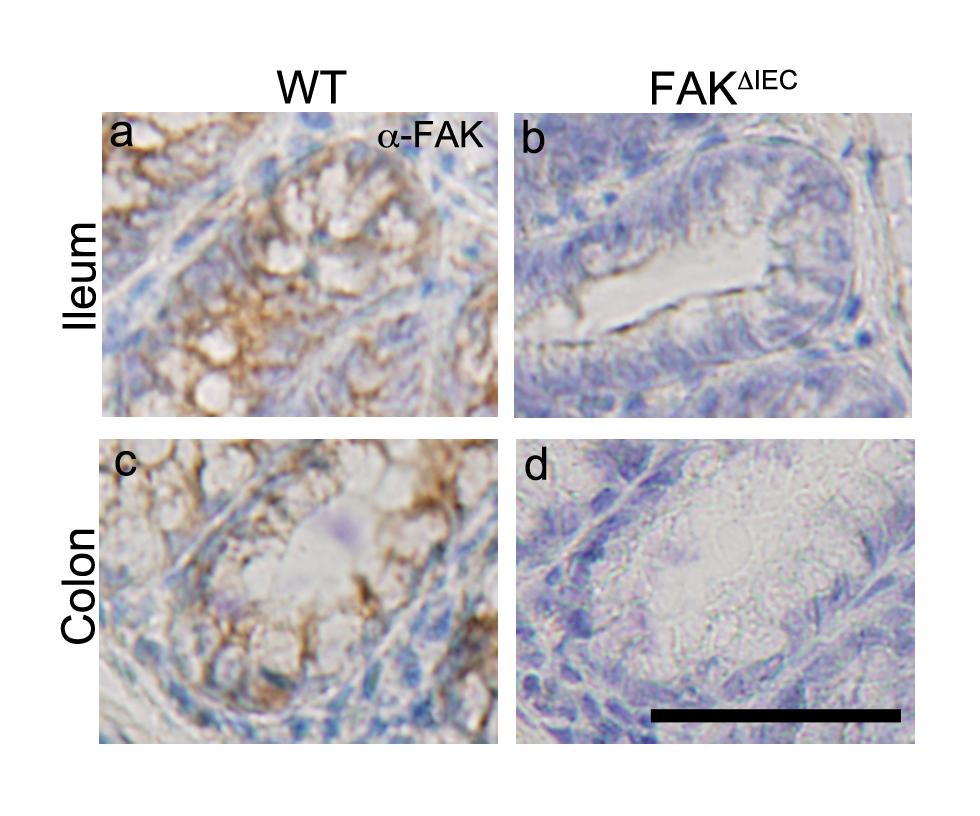

Supplement: Figure S2 — Efficient FAK deletion occurs at the base of intestinal crypts in FAKΔIEC mice. Ileum and colon sections from WT and FAKΔIEC mice were immunostained for FAK. Panels represent an enlarged region from crypts of ileum (a, b) and colon (c, d). Images show positive FAK staining in the base of crypts from WT animals that is absent in crypts from FAKΔIEC animals. Bar represents 50 µm. (TIF) [file pone.0023123.s002.tif]

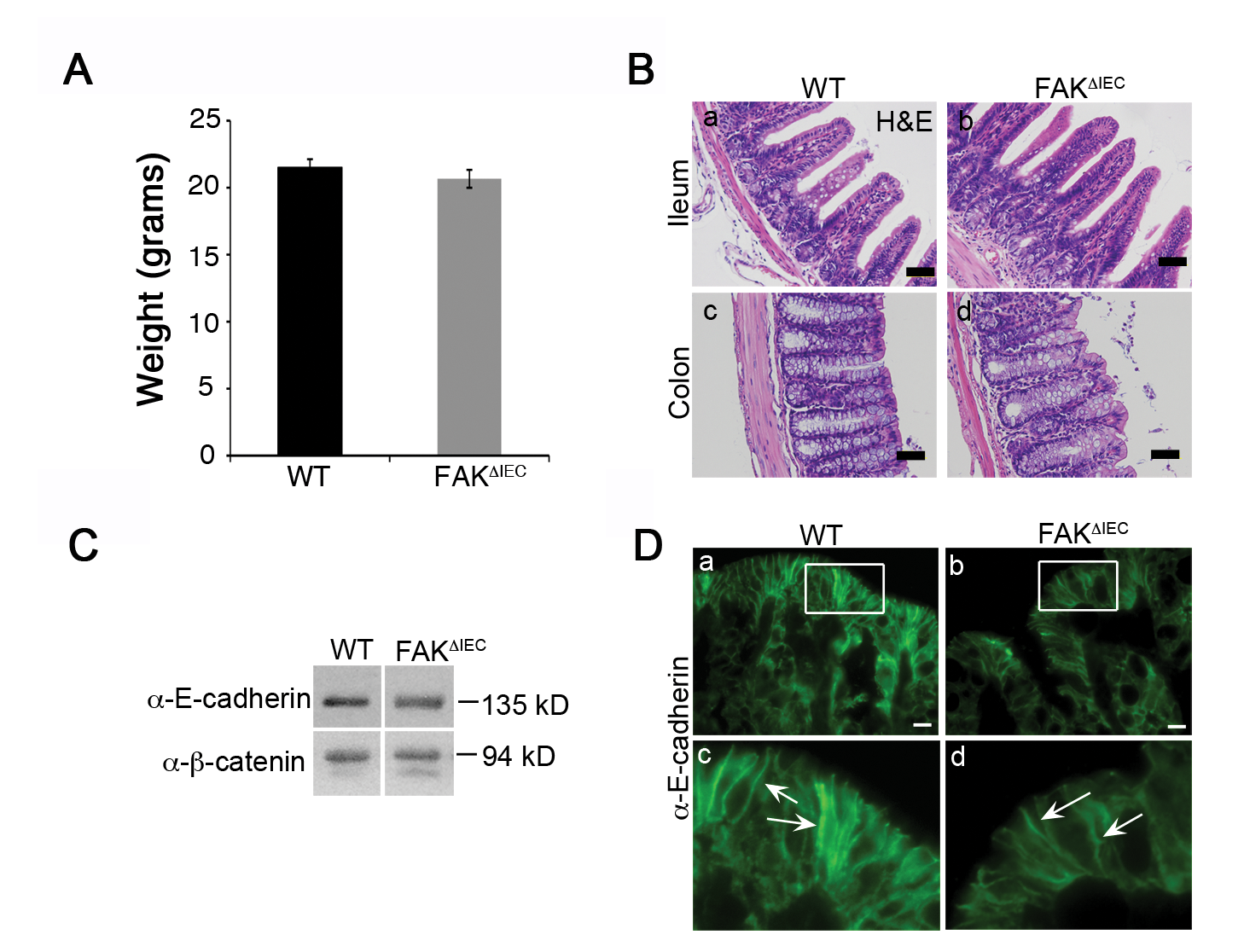

Supplement: Figure S3 — IEC-specific conditional FAK knockout mice maintain normal weight patterns and gut architecture compared to controls. (A) Average body weight in grams of 8–10 week-old WT and FAKΔIEC mice. Data presented are the average of 12 WT and 11 FAKΔIEC mice. (B) H&E stained ileum and colon sections isolated from 8–10 week-old WT and FAKΔIEC mice. (C) Immunoblot analysis of E-cadherin and ß-catenin protein present in primary colonocytes isolated from WT and FAKΔIEC mice. The vertical line separator is indicative of non-contiguous lanes on the gel. However, immunoblots shown for each antibody were generated from a single exposure. (D) Colon sections from WT and FAKΔIEC mice were stained for E-cadherin (green staining) and examined by immunofluorescence. Bars represent 10 µm (panels a, b). Panels c and d represent enlarged regions from panel a and b respectively. Arrows depict regions of membrane-associated E-cadherin staining. (TIF) [file pone.0023123.s003.tif]

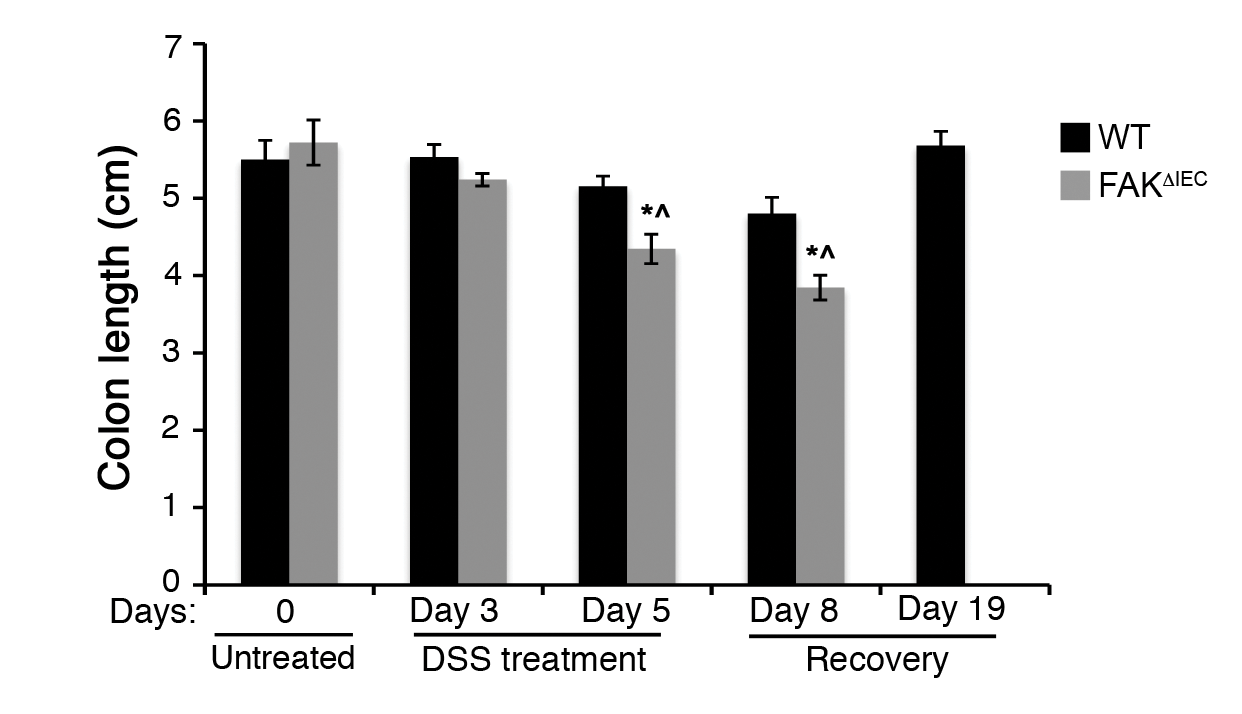

Supplement: Figure S4 — DSS-induced colonic shortening is aggravated in FAKΔIEC mice. Colon length measured in centimeters from untreated and DSS-treated WT and FAKΔIEC mice. Asterisks indicate values that are significantly different from untreated WT mice (Day 0). ∧ indicate values that are significantly different from WT mice at the same time point. In both cases, p<0.05. (TIF) [file pone.0023123.s004.tif]

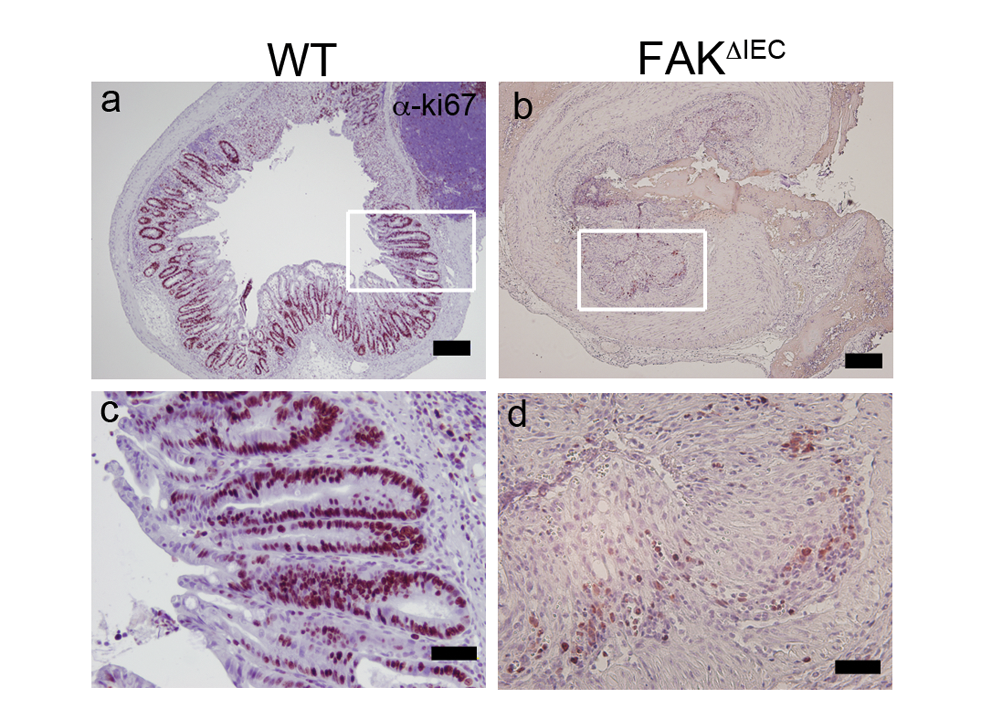

Supplement: Figure S5 — Colons of FAKΔIEC mice generally lack crypt structure at day 8 post-DSS treatment. Low magnification images of ki67-stained DSS-treated colon sections from WT (panel a) and FAKΔIEC (panel b) animals (day 8). Bars represents 200 µm. Panels c and d show high magnification images of panels a and b, respectively. Bars represents 50 µm. (TIF) [file pone.0023123.s005.tif]
