# Supplementary material for: FAK Regulates Intestinal Epithelial Cell Survival and Proliferation during Mucosal Wound Healing
Source: PLoS One. 2011 Aug 24;6(8):e23123. doi: 10.1371/journal.pone.0023123 (PMC3160839; doi:10.1371/journal.pone.0023123)
Supplement: Methods S1 — Genotyping of mice and analysis of Cre-mediated recombination. Animals were genotyped using tail DNA and subjected to PCR analysis. The following primers were used for PCR of the FAK locus: P1 (5′-GAGAATCCAGCTTTGGCTGTTG-3′) and GenoRV (5′-GAATGCTACAGGAACCAAATAAC-3′). This primer set generates 290-bp (WT) and 400-bp (FAKf) products. To determine the status of the villin locus, the following primers were used: MR1878 (5′- GTGTGGGACAGAGAACAAACC-3′) and MR1879 (5′- ACATCTTCAGGTTCTGCGGG-3′). These primers generate an 1100-bp (Villin-Cre) product. For PCR of the ROSA26 allele, the following primers were used: ROSA1 (5′- AAAGTCGCTCTGAGTTGTTAT-3′), ROSA2: (5′-GCGAAGAGTTTGTCCTCAACC-3′) and ROSA3: (5′- GGAGCGGGAGAAATGGATATG-3′). ROSA1 and ROSA3 primer sets generate a 600-bp product containing the WT allele, while ROSA1 and ROSA2 primer sets generate a 300-bp product containing the ROSA26LacZf-STOP-f allele. To check for Cre-mediated recombination in intestinal tissues, DNA was isolated from homogenates of intestinal tissues (ileum, cecum, colon) and subjected to PCR. The following primers were used: LoxP (5′GACCTTCAACTTCTCATTTCTCCC-3′) and GenoRV (see above). These primers amplified products consisting of a FAKf/f (1.6 kb), and a Cre-mediated recombined fragment (327 bp). PCR fragments were separated on 1.5% agarose gels. (DOCX) [file pone.0023123.s006.docx]

**Methods S1: Genotyping of mice and analysis of Cre-mediated recombination.** Animals were genotyped using tail DNA and subjected to PCR analysis. The following primers were used for PCR of the FAK locus: P1 (5’-GAGAATCCAGCTTTGGCTGTTG-3’) and GenoRV (5’-GAATGCTACAGGAACCAAATAAC-3’). This primer set generates 290-bp (WT) and 400-bp (FAK^f^) products. To determine the status of the villin locus, the following primers were used: MR1878 (5’- GTGTGGGACAGAGAACAAACC-3’) and MR1879 (5’- ACATCTTCAGGTTCTGCGGG-3’). These primers generate an 1100-bp (Villin-Cre) product. For PCR of the ROSA26 allele, the following primers were used: ROSA1 (5’- AAAGTCGCTCTGAGTTGTTAT-3’), ROSA2: (5’-GCGAAGAGTTTGTCCTCAACC-3’) and ROSA3: (5’- GGAGCGGGAGAAATGGATATG-3’). ROSA1 and ROSA3 primer sets generate a 600-bp product containing the WT allele, while ROSA1 and ROSA2 primer sets generate a 300-bp product containing the ROSA26LacZ^f-STOP-f^ allele. To check for Cre-mediated recombination in intestinal tissues, DNA was isolated from homogenates of intestinal tissues (ileum, cecum, colon) and subjected to PCR. The following primers were used: LoxP (5’GACCTTCAACTTCTCATTTCTCCC-3’) and GenoRV (see above). These primers amplified products consisting of a *FAK^f/f^* (1.6 kb), and a Cre-mediated recombined fragment (327 bp). PCR fragments were separated on 1.5% agarose gels.
