# Supplementary material for: FAK Regulates Intestinal Epithelial Cell Survival and Proliferation during Mucosal Wound Healing
Source: PLoS One. 2011 Aug 24;6(8):e23123. doi: 10.1371/journal.pone.0023123 (PMC3160839; doi:10.1371/journal.pone.0023123)
Supplement: Methods S2 — Preparation of tissue sections and immunohistochemical staining. Ileum and colon tissues were flushed with PBS to remove fecal material. Tissues were then fixed overnight in Bouin's fixative (Ricca Chemical Company, Arlington, TX), 10% formalin or snap-frozen in O.C.T. (optimal cutting temperature, Sakura Finetek U.S.A., Inc., Torrance, CA). Following fixation, tissues were washed in 70% ethanol. Segments were then embedded in paraffin directly or cut and mounted in agar-10% formalin prior to being embedded in paraffin. Five-micron paraffin sections were cut and mounted onto slides. For immunohistochemical (IHC) staining, slides were deparaffinized in a series of xylene and ethanol baths. Slides were treated in 0.3% hydrogen peroxide to block endogenous peroxidase activity. Antigen retrieval was performed by microwaving slides for 20 minutes in 10 mM sodium citrate buffer (pH 6.0). IHC staining was performed utilizing a biotin blocking kit and Vectastain ELITE ABC kit as per manufacturer recommendations (Vector Laboratories, Burlingame, CA). Slides were incubated with primary antibodies in PBS containing Vector blocking agent. Biotinylated secondary anti-rat, anti-rabbit, or anti-mouse antibodies (Vector Labs) were added and incubated for 10 minutes at room temperature. Sections were then incubated with Nova Red or 3,3-Diaminobenzidine (DAB) substrate (Vector Labs) followed by a hematoxylin counterstain. For analysis of tissue architecture, hematoxylin and eosin (H & E) staining was performed. To visualize connective tissue, Masson's trichrome staining was performed. For immunofluorescence, five-micron frozen sections were cut and mounted onto slides. Sections were blocked and stained in 10% goat serum at room temperature. Sections were incubated with primary antibodies for 1 hour followed by a 30 minute incubation with anti-mouse fluorescein isothyocyanate (FITC)-conjugated secondary antibodies (Jackson ImmunoResearch, West Grove, PA). Cells were imaged with a Niko [file pone.0023123.s007.docx]

**Methods S2: Preparation of tissue sections and immunohistochemical staining**

Ileum and colon tissues were flushed with PBS to remove fecal material. Tissues were then fixed overnight in Bouin’s fixative (Ricca Chemical Company, Arlington, TX), 10% formalin or snap-frozen in O.C.T. (optimal cutting temperature, Sakura Finetek U.S.A., Inc., Torrance, CA). Following fixation, tissues were washed in 70% ethanol. Segments were then embedded in paraffin directly or cut and mounted in agar-10% formalin prior to being embedded in paraffin. Five-micron paraffin sections were cut and mounted onto slides. For immunohistochemical (IHC) staining, slides were deparaffinized in a series of xylene and ethanol baths. Slides were treated in 0.3% hydrogen peroxide to block endogenous peroxidase activity. Antigen retrieval was performed by microwaving slides for 20 minutes in 10mM sodium citrate buffer (pH 6.0). IHC staining was performed utilizing a biotin blocking kit and Vectastain ELITE ABC kit as per manufacturer recommendations (Vector Laboratories, Burlingame, CA). Slides were incubated with primary antibodies in PBS containing Vector blocking agent. Biotinylated secondary anti-rat, anti-rabbit, or anti-mouse antibodies (Vector Labs) were added and incubated for 10 minutes at room temperature. Sections were then incubated with Nova Red or 3,3-Diaminobenzidine (DAB) substrate (Vector Labs) followed by a hematoxylin counterstain. For analysis of tissue architecture, hematoxylin and eosin (H & E) staining was performed. To visualize connective tissue, Masson’s trichrome staining was performed. For immunofluorescence, five-micron frozen sections were cut and mounted onto slides. Sections were blocked and stained in 10% goat serum at room temperature. Sections were incubated with primary antibodies for 1 hour followed by a 30 minute incubation with anti-mouse fluorescein isothyocyanate (FITC)-conjugated secondary antibodies (Jackson ImmunoResearch, West Grove, PA). Cells were imaged with a Nikon Eclipse E800 microscope connected to a charged-coupled device (CCD) camera. Imaging was performed using Openlab software (Perkin Elmer, Waltham, MA, USA). IHC- and H & E-stained sections were examined with an Olympus BX51 microscope and images were acquired with an Olympus DP70 digital camera controlled by Image Pro Plus^TM^ software (EPIX, Inc, Buffalo Grove, IL).
