# Supplementary material for: FAK Regulates Intestinal Epithelial Cell Survival and Proliferation during Mucosal Wound Healing
Source: PLoS One. 2011 Aug 24;6(8):e23123. doi: 10.1371/journal.pone.0023123 (PMC3160839; doi:10.1371/journal.pone.0023123)
Supplement: Methods S3 — Soft-plate96 proliferation assays. To assay cell growth on substrates of increasing rigidity, we employed the use of a multiwell “soft-plate” as described in Tilghman et al. [49]. Soft-plate96 assay plates were seeded with siControl or siFAK-treated cells in sextuplet wells 24 hours post-siRNA transfection at a density of 5000 cells per well, and the cells were allowed to proliferate for a further 48 hours. Cell proliferation was measured using the CyQuant NF cell proliferation assay kit (Invitrogen, Carlsbad, CA). Standard curves were generated for each experiment by performing serial dilutions of the cells in an empty row of wells and allowing them to adhere for four to six hours prior to quantification with CyQuant. (DOCX) [file pone.0023123.s008.docx]

**Methods S3: Soft-plate96 proliferation assays**

To assay cell growth on substrates of increasing rigidity, we employed the use of a multiwell “soft-plate” as described in Tilghman *et al.* [49]. Soft-plate96 assay plates were seeded with siControl or siFAK-treated cells in sextuplet wells 24 hours post-siRNA transfection at a density of 5000 cells per well, and the cells were allowed to proliferate for a further 48 hours. Cell proliferation was measured using the CyQuant NF cell proliferation assay kit (Invitrogen, Carlsbad, CA). Standard curves were generated for each experiment by performing serial dilutions of the cells in an empty row of wells and allowing them to adhere for four to six hours prior to quantification with CyQuant.
